# COVID‐19 and Oncology: Service transformation during pandemic

**DOI:** 10.1002/cam4.3384

**Published:** 2020-08-18

**Authors:** Marco Shiu Tsun Leung, Shangzhe George Lin, Jason Chow, Amer Harky

**Affiliations:** ^1^ Department of Medical Oncology St George’s University Hospital NHS Foundation Trust London UK; ^2^ Faculty of Medicine St George’s Hospital Medical School London UK; ^3^ Department of Cardiothoracic Surgery Liverpool Heart and Chest Hospital Liverpool UK; ^4^ Department of Integrative Biology, Faculty of Life Sciences University of Liverpool Liverpool UK

**Keywords:** chemotherapy, COVID‐19, oncology service, SARS‐CoV‐2, systemic anticancer therapy, telemedicine

## Abstract

**Background:**

The COVID‐19 pandemic has challenged healthcare systems around the world, where resources have refocused on increasing critical bed capacity to prepare for the peak in incidence of COVID‐19. Oncology faces an unprecedented challenge as patients require multidisciplinary care and are more likely to be immunosuppressed. Services in oncology have been transformed using minimal resources over a short period of time. This transformation continues and telemedicine is playing a key role.

**Aims:**

We explore how services in oncology have transformed to deliver services including consultations, systemic anticancer therapy, and surgery for patients, while shielding them from contracting COVID‐19. We assess the risks and benefits of the service transformation in the immediate, interim, and long term, and how telemedicine supports the process.

**Methods:**

We performed a comprehensive review of the literature using suitable keywords on the search engines of PubMed, SCOPUS, Google Scholar, and latest official data from May to June 2020.

**Results:**

Through the published literature on this topic, we discuss the transformations in oncology and the impact on patients and healthcare workers due to the COVID‐19 pandemic. We reflect on the lessions from COVID‐19 and assess the role of telemedicine in the future of oncology services.

**Conclusion:**

Transformation of services in oncology effectively shields patients from COVID‐19 infections, and telemedicine plays a role in virtual consultations. The long‐term effects are yet to be seen, such as safety of home‐based treatment, and effectiveness of virtual communication on patient care. As oncology requires a multidisciplinary approach, telemedicine will play a key role to improve patient‐centered cancer care in the future.

## Graphical abstract

Cancer service is affected severely during COVID‐19. Transformation of health service to meet patient demands in cancer should be utilized to provide safe and effective care

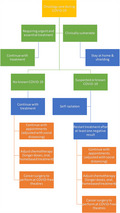

## 1 INTRODUCTION

**Figure 1:**
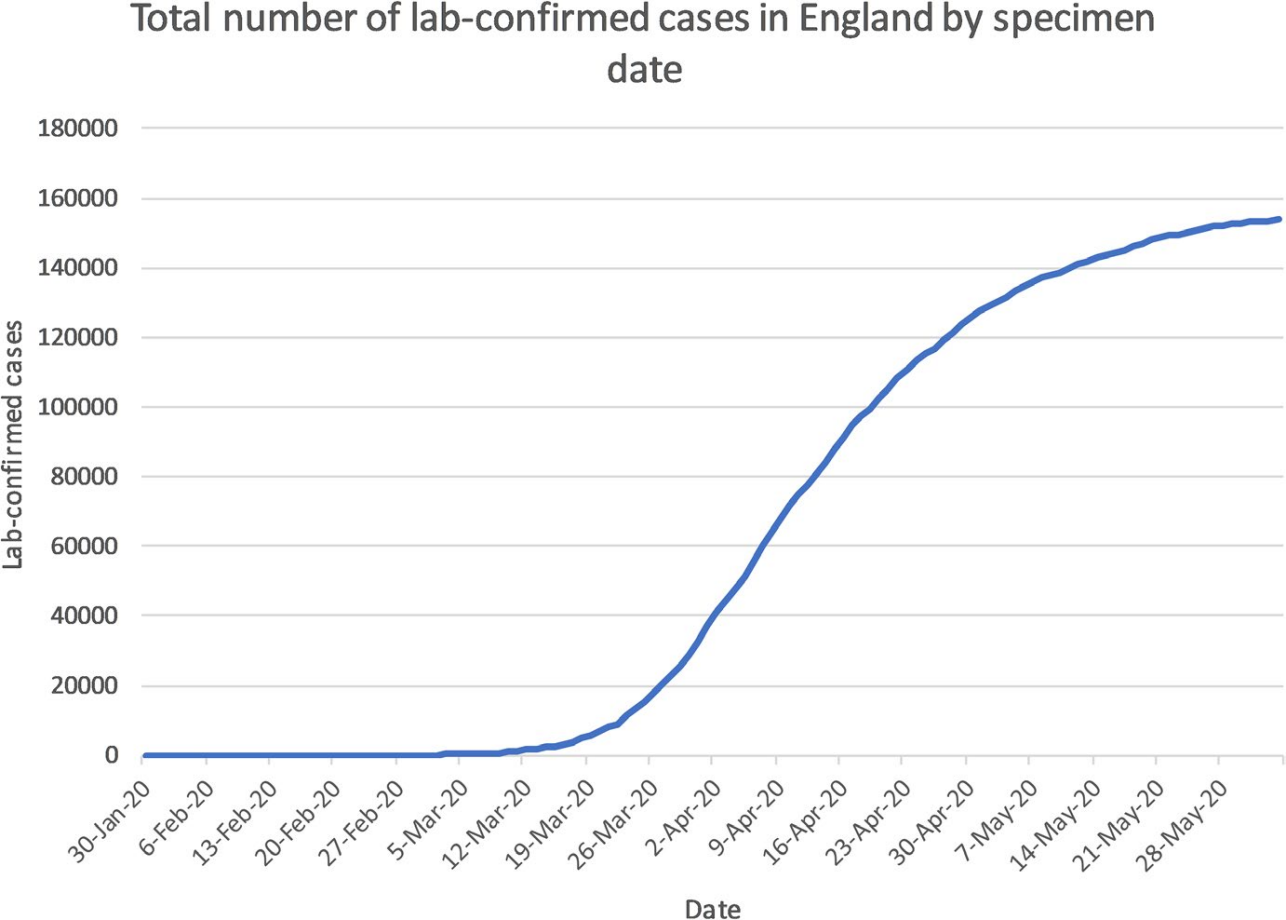
Graph shows total number of lab‐confirmed cases of COVID‐19 in the UK by date. It recorded the number of confirmed cases from late January to over 150 000 cases by June 2020^4^

**Figure 2:**
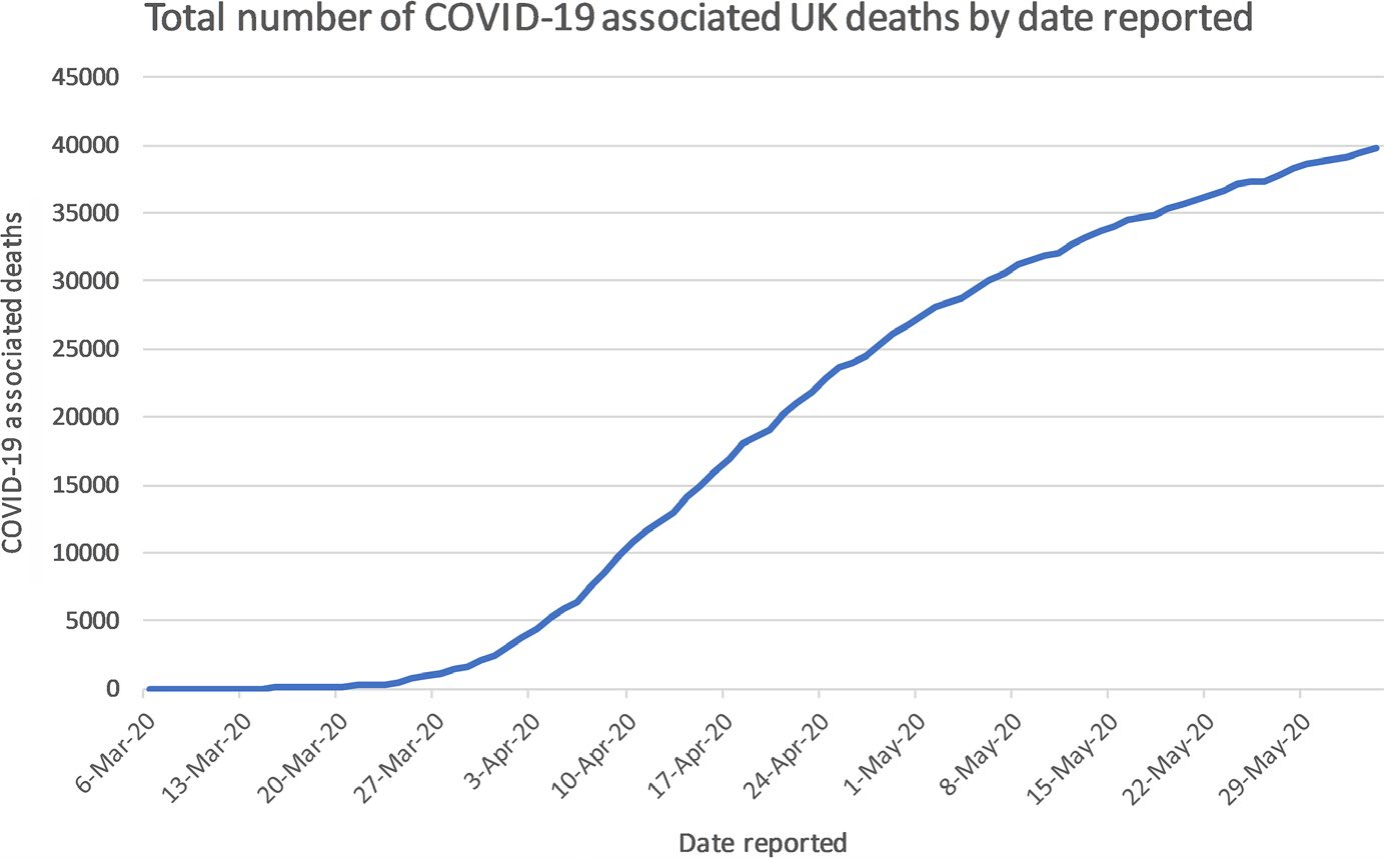
Graph shows the recorded number of deaths from March 2020 to June 2020 in the UK. It recorded several deaths since early March to over 39 000 deaths by June 2020 ^3^

The emergence of the novel severe acute respiratory syndrome coronavirus (SARS‐CoV‐2) pandemic has become a global health challenge, which was firstly discovered in China in late 2019.^1^ Coronavirus disease (COVID‐19) causes fever, dry cough, dyspnea, loss of smell, and other nonspecific symptoms, meaning it is difficult to differentiate COVID‐19 from other infectious diseases.^2^ Due to its high transmission risk, it has affected almost six‐million people causing 382 867 deaths in almost all continents as of 5 June 2020. And in the United Kingdom (UK), it has claimed over 39 000 deaths (see Figure 2.) of those 153 807 lab‐confirmed cases (see Figure 1.).^3^, ^4^

In response to the COVID‐19, the UK government's coronavirus action plan was launched in early March and initiated a locked down since late March 2020.^5^, ^6^ The government's plan is to protect National Health Service (NHS) from being overwhelmed from the exponential growth in COVID‐19 confirmed cases. The NHS transformed their services to maximize inpatient and critical care capacity to free up a target of at least 30 000 beds of the 100 000 beds for COVID‐19.^7^ In the immediate period, all services including oncological services were impacted while resources were refocused on the government's COVID‐19 response. At the same time, the NHS experienced an unprecedented staffing crisis due a significant number of staff being infected and/or in self‐isolation.^8^, ^9^ Due to the service transformation or patients’ personal choice, there was on average a fall of 30% of patients receiving chemotherapy and up to 50% fall in new patients presenting or referred for suspected cancer diagnosis in March to April 2020.^7^

In the interim period, essential and urgent cancer services continued, whereas referrals and other services were postponed or transformed to enable virtual delivery.^10^ Because patients with cancer are more likely to be immunosuppressed, it is important minimize their risk of exposure to SARS‐CoV‐2. Strategies include requiring staff and patients to wear personal protective equipment (PPE) to prevent viral transmission or setting up home delivery of certain systemic anticancer therapy (SATs), such as home delivery of tyrosine kinase inhibitors (TKIs) or home delivery of Herceptin, given subcutaneously.^11^, ^12^, ^13^ Home delivery of certain SATs enables patients to avoid unnecessary visits to hospitals and reduces waiting time for other patients on the chemotherapy suite, thereby allowing better social distancing.^14^ To guide clinicians to make better decisions, National Institute for Health and Care Excellence (NICE) has developed a triage system to categorize patients by their odds of survival from treatment ^15^ (Table 1). For patients requiring treatment at hospital, such as surgery, they are referred to the newly developed COVID‐free cancer hubs for procedures to be performed (Table 2). Furthermore, long‐term effects are unknown, such as the challenge to maintain social distancing, safety of prolonged home‐based treatment, and safety issues surrounding the ability to remotely monitor drug toxicities.

**Table 1:** Prioritization system for patient receiving systemic anticancer treatment

		Priority 1	Priority 2	Priority 3		Priority 4	Priority 5	Priority 6
Curative treatment	Chance (%) of success for curative treatment	>50%	20%‐50%		10%‐20%	0%‐10%	—	—
Adjuvant or Neoadjuvant treatment	Additional chance (%) to cure	>50%	20%‐50%		10%‐20%	<10%	—	—
Non‐curative treatment	Chance of immediate extension of life of 1‐year or more.	—	—		>50%	15%‐50%	>50% (chance of palliation or temporary tumor control)	15%‐50% (chance of palliation or temporary tumor control)

**Table 2:** Prioritization system for patient receiving cancer surgery

	Priority 1a	Priority 1b	Priority 2	Priority 3
Type of surgery	Emergency—operation needed with 24 hours to save life	Urgent—operation needed with 72 hours	Elective—operation needed within 4 weeks to save life/progression of disease	Elective—can be delayed for up to 10‐12 weeks without having predicted negative outcome

The future development of oncological service following the COVID‐19 pandemic is unknown, but there is an increasing demand for virtual medical services—where the current technological capabilities remain mostly on telephone consultations in the NHS. Furthermore, it is important to provide support for healthcare workers’ physical and mental health as the NHS plans for the long‐term service transformation.^16^

## 2 COVID‐19 

### 2.1 Origin and emergence of COVID‐19

The phenomenon of coronavirus‐based epidemics was first seen in 2002‐2003 when cases of atypical pneumonia were identified in Guangdong Province, China, later spreading to Hong Kong where researchers were able to isolate the pathogen and classified it as a coronavirus, naming the resulting disease severe acute respiratory syndrome (SARS). International travel subsequently spread SARS to a further 26 countries, resulting in a case load of more than 8000 with an approximate 10% fatality rate.^17^ Another example of a coronavirus from an animal origin that resulted in a much publicized epidemic is the Middle East respiratory syndrome coronavirus (MERS‐CoV) which emerged in 2012 and was characterized by a much higher case fatality rate, but limited human‐to‐human transmission.^18^

The initial chronology of the COVID‐19 pandemic is controversial, but the following is one of the commonly accepted chronology. The first reported cases of the novel coronavirus were identified in late December of 2019; from 18 to 29, five patients were hospitalized with one case ending in death.^1^, ^19^ The origin of the identified cases was epidemiologically traced back to the Huanan seafood and wet animal wholesale market in Wuhan, Hubei Province, China.^20^ By 25 January 2020, cases in the Chinese mainland had progressed to a total of 1975 cases and 56 deaths.^21^

### 2.2 Global spread

The World Health Organization (WHO) first declared the outbreak of COVID‐19 as a Public Health Emergency of International Concern (PHEIC) on 30 January 2020,^9^ further recognizing it as a pandemic on 11 March 2020.^22^

According to the WHO’s latest situation report on 4 June 2020, the global confirmed case count sits at 6 416 828 cases and a confirmed death count of 382 867 deaths, with confirmed cases in all continents excluding Antarctica; as the count reflects only lab‐confirmed cases and deaths the actual figures for both counts may be significantly higher, especially in developing countries where testing is not as easily accessible and implemented. The United States has the highest global count in both confirmed cases and deaths by a majority, with the UK following behind in terms of deaths but not cases.^3^, ^4^

### 2.3 Epidemiology of COVID‐19 in the UK

The first recorded case of COVID‐19 in a British national is thought to be Connor Reed, a 25‐year‐old Welsh national working at a college in Wuhan, China,^23^ he remained in Wuhan throughout his diagnosis and disease course. In May 2020, the BBC reported the experiences of Dr John Wright of Bradford Royal Infirmary in encountering COVID‐19‐like symptoms in members of a choir in Yorkshire returning from a business trip on 17 or 18 December, long before the first confirmed cases.^24^

The first confirmed cases in the UK were diagnosed in the week commencing 27 January 2020, both were identified as at risk while still in the community and were transported from their hotel to the Infectious Diseases Unit at Hull University Hospitals where they were managed in separate negative pressure rooms until results were available, after which they were transferred to the High‐Consequence Infectious Diseases Unit (HCID) in Newcastle, UK. The third confirmed case was reported on 6 February 2020, a Brighton man who returned from Singapore and France on 28 January.^25^ By 12 March, the UK case total had risen to 460,^26^ which combined with the WHO’s PHEIC declaration provoked the escalation of the risk level in the UK from moderate to High.^27^ As of 5 June 2020, the statistics for COVID‐19 in the UK are as follows, 153 807 total lab‐confirmed cases (see Figure 1.) and 39 728 confirmed deaths (see Figure 2.), giving an infection fatality rate of approximately 14.1%.^3^

The UK government's coronavirus action plan was launched on 3 March,^27^ further listing it as a notifiable disease on 5 March^6^ and initiating lockdown on the 23 March.^5^ As of 30 May, the government is slowly moving back into a phase of loosening lockdown restrictions and reopening shops, services, and schools in a staggered stage model.^28^

## 3 COVID‐19 AND CANCER

### 3.1 Incidence and prevalence

Although the evidence base is limited, a cross‐sectional study based in Wuhan, China found that a sample size 1524 cancer in‐patients over a 6‐week period had a 0.79% infection rate, higher than the 0.37% cumulative incidence in the full hospital patient community, although all these oncology in‐patients were in a poor enough condition to warrant admission which in itself is a poor prognostic factor for infection risk, as well as the fact that community cancer patients were unaccounted for.^29^

### 3.2 Presentation

Clinical characteristics in cancer patients with COVID‐19 do not differ to the non‐cancer population and includes the following and summarized in Table 3.

‐ Fever
‐ Dry cough
‐ Dyspnoea
‐ Chills
‐ Muscle pain
‐ Headache
‐ Sore throat
‐ Rigors
‐ Loss of taste and/or smell ^2^

**Table 3:** Common clinical features in patients with COVID‐19 regardless of cancer status, as there is very little data on clinical feature incidence specific to the COVID‐19 patient population with cancer.

Clinical Features	Incidence	Study Population	Additional Notes
Fever	99%	138 COVID‐19 positive in‐patients in a single hospital in Wuhan, China ^29^	Fever is not a universal finding on presentation, in one study approximately 20% of patients had a low‐grade fever of < 38°C,^2^ and in another study fever was present in 44% on admission and ultimately 89% during hospitalization.^30^
Fatigue	70%		Limited data on these clinical features
Dry cough	59%		
Anorexia	40%		
Myalgias	35%		
Dyspnea	31%		
Sputum Production	27%		
Smell and/or taste sensory alterations	64%	202 COVID‐19 patients with mild symptoms suitable for home management in Treviso and Belluno, Italy ^31^	24% of patients (out of total population) reported severe sensory loss.^31^ However, these statistics were self‐reported and as such objective rates of sensory loss may be much lower, in another study only 62% of the 86 patients who had total lack of smell had an objectively measurable loss^32^
Gastrointestinal symptoms	18%	4243 total patients from a meta‐analysis of 60 studies, populations ranging from China, South Korea, Singapore, Vietnam, United States, UK^33^	Diarrhea (13%), nausea/vomiting (10%), abdominal pain (9%)^33^

The incidence figures therefore cannot be fully applicable to the COVID‐19 cancer patient population but can be used as a rough guidance.

Data are limited, however, with most existing studies limited by small sample sizes. A report consisting of 28 COVID‐19 patients from three hospitals in Wuhan, China showed that the most common characteristics were fever, dry cough, lymphopenia, and anemia with 75%‐82% of patients presenting with these; 54% of patients had severe disease, and 21% required ICU admission; severe events were more common in patients who had chemotherapy, radiotherapy, targeted therapy, or immunotherapy in the last 14 days; patch consolidation on CT scan was associated with greater risk of severe disease.^30^

### 3.3 Prognosis

COVID‐19 presents with a higher mortality rate and severity in an older population; regardless of age patients with a pre‐existing medical condition are also at increased risk if infected.^31^, ^32^

Developing data suggest that an increased severity of COVID‐19 disease course is associated with adult patients with cancer.^2^

Early data from Wuhan, China in an analysis of 105 patients whose outcomes were matched with 536 control patients showed that in the cancer population, Lung cancer was the most frequent malignancy followed by gastrointestinal, breast, or thyroid, then hematological malignancy; compared with the noncancer control population, cancer patients had higher death rates, ICU admission rates, a greater likelihood of severe symptoms and a doubled chance of requiring invasive mechanical ventilation; however, cancer patients also were more often smokers and experienced more in‐hospital infections, which may have been contributing factors to the statistical differences.^33^

## 4 IMPACT ON ONCOLOGICAL SERVICES

### 4.1 Immediate impact on oncological services

In response to the COVID‐19 pandemic, the National Health Service (NHS) had experienced an unpresented challenge. Over a very short period of time, NHS had to adapt their services to meet the demands of the sudden surge of patients presented with COVID‐19 symptoms. The limited data and misinformation had left frontline health workers in frustration to adapt their services effectively.^34^

Retrospectively in March 2020, the NHS was hit by one of the worst staffing crises.^7^, ^8^ This was due to several factors including the global limited access to personal protective equipment (PPE), medical supplies, and upsurge of healthcare workers suffering from COVID‐19.^35^ The WHO advises to protect healthcare workers as one of the priorities meant the threshold of a staff required to be in isolation is very low. Any staff presenting any symptoms of COVID‐19 are required to self‐isolate.^9^ This not only had an immediate impact on the service capability of the NHS, but also long‐term effects on the individual healthcare worker, which is discussed further below.^16^ This has further been exacerbated by the limited COVID‐19 testing capacities, which was only at less than 1000 tests performed per day in late March.^36^ Moreover, the NHS experienced one of the largest redeployment of staff to redistribute the workforce in order to meet the demands of the coronavirus pandemic.^37^ Some local oncology teams had seen over half of their team redistributed to COVID‐19‐related duties.

Concurrently, there was a sharp drop in the number of patients receiving oncological services. Due to the priority to maximize inpatient and critical care capacity with targets of freeing up over 10 000 hospital beds across England.^7^ Along with similar strategies, it had led to a 30% reduction in patients receiving chemotherapy, which included both who are asked to be shielded from hospitals, and those patients who voluntarily postponed their treatment. Due to the suspension of certain GP practices, the department experienced a fall in over 50% presentation of new patients referred from general practitioners.^7^

### 4.2 Impact on services during the interim period

Following the immediate period, the NHS swiftly developed mechanisms to better balance the services that were targeted to fight against the pandemic, but also to retain essential and urgent non‐COVID services.

In this interim period, the senior leadership of NHS Cancer services released a letter in end of March to advise on cancer treatment in response to COVID‐19 pandemic.^10^ The key recommendations included, first, essential and urgent cancer services must continue, and discuss with patients about risks associated with continuing treatment, second, referrals depart from normal practice and that safety netting must be in place to allow patients to be followed up, and, third, the development of COVID‐free hub for cancer surgery with a centralized triage system.^10^ These key pillars serve to guide the decision and changes made by Clinical Commissioning Groups (CCGs), Hospital Trusts and local hospital.

While these changes took place, it allowed an alleviation in the shortage of staff as some return to work. Yet, many staff members were redeployed to COVID wards, while those who are in isolation are encouraged to conduct telephone and video consultations with patients. NICE also advises to support staff who are in self‐isolation through frequent contact to protect their mental wellbeing.^38^ Studies have explored that self‐isolation can lead to making people feeling anxious and unsafe, of that like acute address disorder, where it is likely to continue following quarantine.^16^

Like other specialties, care in oncology is delivered with consideration of COVID‐19: If a patient is *not known* to have COVID‐19, NICE has recommended that patients are to attend appointments by themselves without family members or carers as this would reduce the risk of contracting COVID‐19. When patients attend their appointment, service providers can reduce time spent at the waiting area of the hospital or clinic through effective scheduling. Another strategy involves asking patients to wait outdoors or in their cars and inform them text if application when the Doctor or healthcare worker is ready to see them.^11^

At our local oncology department, the wards are divided into two types, where one is for those patients who are free of COVID‐19, and the other wards is for those patients who are confirmed or suspected to have COVID‐19. Staffing is also managed to ensure there is no mixing between the clean and dirty teams.

Significant interim changes were implemented to allow for greater flexibility with the aims to maximizing benefits and minimizing risks to patients. SATs can be administered at home or similar setting that reduces patient's exposure to SARS‐CoV‐2. In our department, we applied a similar principle to reduce the number of required visits by prescribing every four weeks, instead of fortnightly, treatment of durvalumab for patients with non‐small lung cancer. This is a suggested protocol from NHS England even though durvalumab is only licensed for up to fortnightly dose.^12^ This strategy reduces the frequency of visits to hospital, in attempt to reduce risk of exposure to the virus. Another recommendation is to stop maintenance chemotherapy, usually given in combination with maintenance immunotherapy, and allowing only immunotherapy to continue.^12^ This may potentially reduce efficacy but improve safety and similarly reduce the risk of toxicity and in turn risk of infection and avoidable hospital visits. Alternatively, chemotherapy has been also recommended to switch to oral therapy to be administered at home, again this reduces the frequency of hospital visits.^12^ However, there is conflicting evidence on immunotherapy, as some argue it mitigates with patients’ risks of contracting the virus or becoming serious ill from COVID‐19, with exceptions to ibrutinib and a tyrosine kinase inhibitor.^13^

Another common practice includes prescribing prophylactic daily granulocyte‐colony stimulating factor (G‐CSF) to prevent the risk of neutropenic fever, thereby reducing admissions. This has been in practice in our local department since late March 2020.^12^ Other changes in oncology treatments developed by the National Comprehensive Network may help guide other oncological departments in their service adaption.^39^

If a patient is *known* or *suspected* to have COVID‐19, patients are advised to follow the most update to date government guidelines on social distancing and self‐isolation. Unless treatment is deemed to be urgent and essential, then patient would be offered to continue with treatment. Otherwise, it is recommended that these patients defer all anticancer treatment until at least one negative test according to NICE.^40^ This contrasts with most other recommendations which generally suggest a minimum period of 14 days from COVID‐19 symptom onset or relocation of treatment from hospital to home.^41^

### 4.3 Prioritizing patients and centralized triage systems

**Figure 3:**
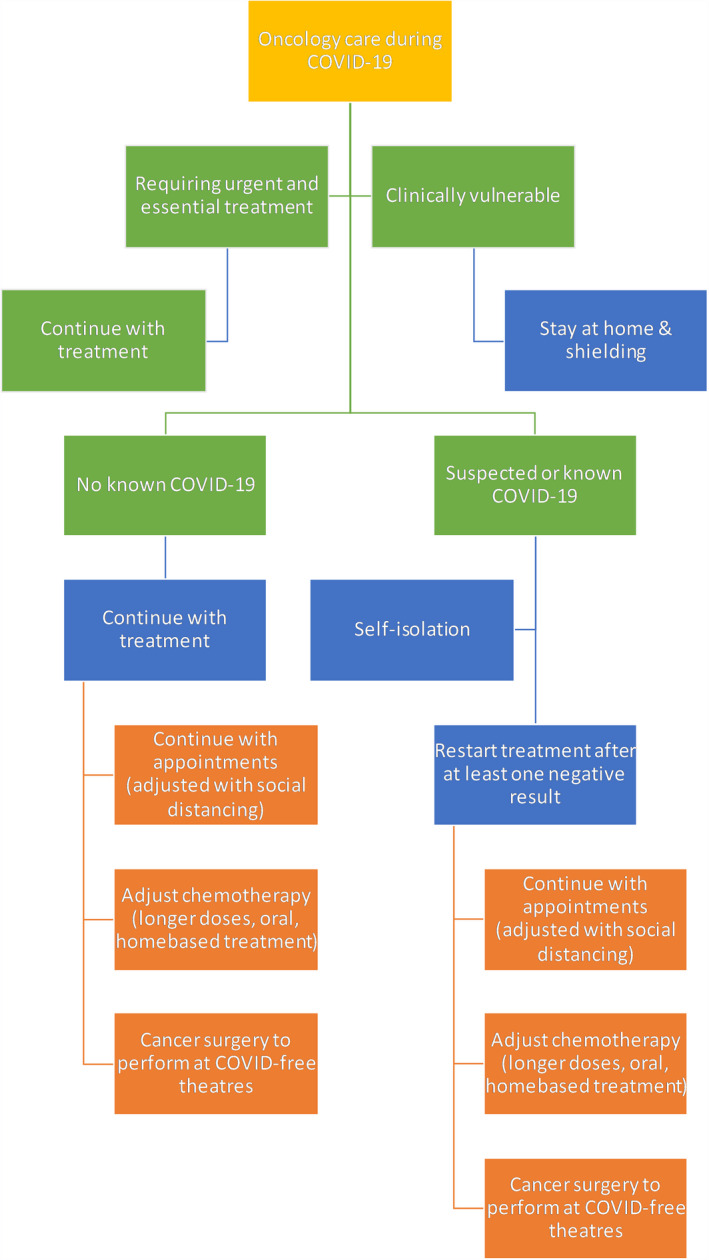
Flow diagram shows the treatment pathways and algorithm for patients receiving oncology services during COVID‐19 pandemic. Recommendations provided by National Institute for Health and Care Excellence (NICE) and National Comprehensive Cancer Network (NCCN)^11^, ^12^, ^38^, ^39^

If resources in NHS become scarce in this COVID‐19 pandemic, it is important to develop clear guidance and a system to prioritize patients based on clinical need. It is also important to develop effective pathways to allow for rapid and efficient triage of patients down the correct diagnostic and treatment pathways. This system should only be used if resources become extremely limited, and decision has to be made to allocate resources to those patients who are in most need ‐ otherwise patients should be provide all available treatment options. Summary of the algorithm is illustrated in Figure 3.

For SATs, NICE developed a six‐tier system to classify patients who require SATs to aid clinicians to make the most appropriate decisions.^15^, ^42^ Details of the prioritization system can be found in Table 1. For example, though a patient with metastatic cholangiocarcinoma considering SATs would fall into priority category 4 or 5.^15^ However, if the patient is fit and well prior to the cancer diagnosis, he may well want to consider SATs and may not have the opportunity to defer treatment for 6 months due to the potential risk of a rapid deterioration in symptoms and quality of life. A clear and informed decisions need to make with the patient as to the benefits and risks of starting palliative chemotherapy. Surgical services smilarly had to adopt in order to cope with the pressures of COVID‐19 infected patients, including orthopaedics, cardiac and cancer surgery.^43^, ^44^ For patients requiring cancer surgery, NICE has developed a similar categorization system to aid surgical decision and planning.^42^ Details are illustrated in Table 2.

Furthermore, these guidelines are to be implemented with consideration of patient priorities as well as the hospital or system's priorities. Similar systems have developed in different countries with similar principles to reduce risk of infection and optimize oncological treatment options for patients. This is paramount of support the patient's emotional wellbeing by providing psychological support as well as the medical care they require.

### 4.4 Long‐term impact on oncological services

The implications on COVID‐19 on the world are unprecedented and have created novel challenges that all healthcare systems around the world must face. There will clearly be long term and permanent changes that will be made to shape how we practice medicine in the future.

One of the major challenges is to maintain social distancing, which is likely to stay as a government advice for months and even years to come. This poses a challenge to face‐to‐face healthcare delivery. The maintenance of social distancing may not be possible in various settings, such as in waiting rooms and when performing common procedures. This will also be a challenge as those requiring frequent visits to hospital are often also those who are clinically extremely vulnerable.^44^

An effective strategy that permits access to services while shielding these vulnerable patients is a priority of oncological teams across the NHS. As highlighted earlier, staff has been encouraged to conduct more virtual practice; however, despite the recent initiatives to digitize services, the NHS remains decades behind in its technological capabilities.^45^ In our oncology department, telephone remains the only option for virtual consultations, while online conferencing software are available on most Trust computers—the department does not have access to any webcam nor any audio devices, such as headphones.

While the NICE Guidelines recommend interim adaptation of anticancer treatments, it is likely these may implement for an extended period, or reintroduced for any subsequent coronavirus outbreaks, which is highly likely during winter. Limited evidence is available on the risks and toxicity of these interim treatment regimes. This leaves a challenge for clinicians who will need to be able to weigh up the best treatment options based on limited data and their own clinical experience in order to best guide patients along their treatment pathways. This is a clear need and emphasis to move treatment to home‐based settings, there are raising concerns on patient safety, as monitoring toxicities from treatment will have to be done more remotely. Delivery of home SATs certainly has its benefits but is not without challenges, and the advent of the global pandemic has expedited these changes into clinical practice, but it will certainly require close monitoring to ensure there is no increased risks to patients’ health and wellbeing.

Another significant impact that is expected to happen with cancer services are the backlog of referrals that were delayed or interrupted due to the onset of the pandemic. For example, at our local cancer center, it is estimated that there are around 2000 outstanding endoscopies (for suspected gastro‐esophageal cancers) had been requested prior to and during the pandemic. With a conversion rate of around 6% to a cancer diagnosis for these referrals, this equates to an approximately 120 potential cancer patients who will receive a cancer diagnosis in the near future and require treatment.^46^ There are also the cohort of patients who have developed symptoms which are suspicious of cancer during the pandemic but have not yet presented to hospital due to concern of COVID19 infection. Further work needs to be done to increase education and public awareness and to encourage patients to attend hospital should they feel unwell, least these patients present too late to be able to receive a diagnosis and treatment.

## 5 SERVICES ADAPTATIONS AFTER COVID‐19 ERA

From the experiences and challenges that NHS both as an organization and as individual staff have faced during the COVID‐19 pandemic, several lessons can be learnt that would help in the modernization and optimization of the NHS.

There has been a widespread implementation of telemedicine with a dual purpose of compromising for the lack of face‐to‐face appointments, as well as optimizing resources where redistributing to where it is most needed—in handling the pandemic.^47^, ^48^ What we can learn from this experience is that the benefits of telemedicine that we have found during this period could also be applied during a time period where COVID‐19 is not present. The use of virtual consultations would allow patients that, after triage were determined to be of lower urgency, to be seen and have any concerns addressed in a much more time and resource efficient manner, and in turn free up those resources for use of patients with a higher urgency issue while also addressing the longstanding problems of over‐booking, delayed, and rescheduled appointments ^49^.

The pandemic has expedited the implementation of many systems in order to reduce the burden on the NHS and to comply with social distancing guidelines, examples of which are telemedicine and virtual clinics mentioned above, as well as work‐from‐home set ups for clinicians. This reflects a prioritization of resource allocation that was needed to achieve this, from which we can also extend to the post‐COVID era in order to streamline and optimize the logistical infrastructure of the NHS.

While the importance of the mental health of healthcare workers has come increasingly into the spotlight in recent years, the COVID‐19 pandemic has further highlighted the need for adequate support as healthcare workers are severely overworked and exposed to an unprecedented level of stress. This can be due to pure physical and mental exhaustion, handling many patient deaths as well as the gap in supply and demand causing difficult decisions in resource allocation to patients, causing moral injury.^16^, ^50^ Sufficient support during the pandemic is sorely needed as well as adequate after care to prevent staff burnout and possible instances of survivor's guilt, to form a “meaningful rather than traumatic narrative”.^51^ The increased awareness from this period will also serve to encourage clinicians and healthcare staff to seek mental health support as well as hopefully serving to advance the existing staff mental health support infrastructure.

## 6 LESSONS FROM COVID‐19

In the specific case of the future optimization and adaptation of oncological services using lessons learned during the COVID‐19 era, the previous aforementioned implementation of telemedicine is particularly relevant as many of the oncology department's patients are immunosuppressed and vulnerable to infection,^52^ and this would also extend to a COVID‐19 absent setting, if at a lesser severity due to treatment and the nature of cancer as a disease.^53^

Beyond infection risk, many patients can be suffering from frailty and severely reduced mobility, making the reduced need for transport a relief, both on the patients and on the health service as an area of resource drain.

The remote delivery of cancer treatment and patient monitoring can also be carried over to the post COVID‐19 era to optimize patient wellbeing as well as the psychological and mental health benefits of reduced hospital visits.^54^

These transformations are not without their limitations. Telemedicine while providing convenience and resource optimization in some areas, is limited in its ability to match certain aspects of in‐hospital, person‐to‐person clinics, including physical examination, establishment of patient‐clinician rapport on the basis of body language as well as staff team relationships and teamwork utilization.^45^ Remote delivery of services also limits the patient's options of treatment and may increase NHS resource drain in certain cases. For the adaptations the NHS has made during the COVID‐19 era to continue to benefit service, clinician, and patient, these limitations must be explored and overcome.

Weaknesses of the existing system have also been exposed by the strain of the pandemic. While some have been compensated for by remote service delivery, others such as the delay of referrals and investigations have not been. In oncology, where diagnosis and treatment are extremely time sensitive due to progression of disease, improved methods of screening, both clinicians executed or patient executed, must be developed and implemented. These could be as simple as increased education on cancer symptom monitoring and self‐assessment or could extend to changed protocol in carrying out investigations to ensure a minimized infection risk for vulnerable patients.

## 7 CONCLUSION

The COVID‐19 pandemic had transformed the oncological services by the principle of patient safety by shielding patients from contracting the virus and spreading to others. This continuing service transformation can be achieved by moving services into virtual format or home‐based treatment. The current access to technology is low that would need a radical change to improve communications with patient. Oncology is multidisciplinary by practice; therefore, many aspects of the service remains only possible or most optimally delivered though face‐to‐face.

## CONFLICT OF INTERESTS

The authors have no conflicts of interest to declare.

## AUTHOR CONTRIBUTIONS

All authors contributed to this paper and have approved the final version of the manuscript.

## ETHICAL APPROVAL

None was obtained as no patient data was involved in our review.

## Data Availability

All data are included in the manuscript, no supplements are available or required.
